# Neurodevelopmental Disorders in Sub‐Saharan Africa: A Survey on Primary Healthcare Workers' Knowledge

**DOI:** 10.1002/hsr2.70760

**Published:** 2025-04-29

**Authors:** Noah Agbo, Yetunde Adeniyi, Onoja Matthew Akpa, Olayinka Omigbodun

**Affiliations:** ^1^ Centre for Child and Adolescent Mental Health University of Ibadan Ibadan Oyo State Nigeria; ^2^ Department of Psychiatry College of Medicine University of Ibadan Ibadan Nigeria; ^3^ University College Hospital Ibadan Nigeria; ^4^ Centre for Early Development, Learning and Care Ibadan Nigeria; ^5^ Daisy Atlantic Schools Ibadan Nigeria; ^6^ Department of Epidemiology and Medical Statistics College of Medicine University of Ibadan Ibadan Nigeria; ^7^ College of Medicine University of Ibadan Ibadan Nigeria

**Keywords:** children and adolescents, knowledge, neurodevelopmental disorders, primary healthcare workers

## Abstract

**Background:**

Neurodevelopmental disorders are a set of conditions that appear early in a child's development, usually before they begin school, and are likely to impair personal, social, academic, or occupational functioning. These conditions are the result of disturbances in brain development caused by genetic, environmental, or rather unknown causes. With the increasing prevalence of neurodevelopmental disorders in low‐ and middle‐income countries such as Nigeria, it is imperative to understand the level of knowledge of primary healthcare workers who, by virtue of being nearest to the people in the community, are the first point of contact for individuals and families seeking healthcare in the community. This study aimed to assess primary healthcare workers' knowledge level about children and adolescents with neurodevelopmental disorders in Abuja, Nigeria.

**Methods:**

This was a cross‐sectional study where 274 primary healthcare workers (mean 39.8 ± 10.1 years) were recruited from 17 urban and rural primary healthcare centers (PHCs) in Abuja, Nigeria. The Modified Knowledge and Attitude Towards Child and Adolescent Mental Disorders questionnaire was employed to examine knowledge of and attitudes towards children with disorders (ASD, ADHD, and ID), and the data obtained were analyzed. Percentages and frequencies were used to describe the socio‐demographic characteristics of the respondents as well as the knowledge of the respondents in the study. Means and standard deviations were used to present continuous data, while Chi‐square was used to investigate the association between categorical variables.

**Results:**

Results from the analysis showed that even with longer working experience of 12.4 ± 6.6 years, respondents had poor knowledge of the identification and management of children and adolescents with neurodevelopmental disorders. A significant majority (68.2%) of the respondents agreed that neurodevelopmental disorders in children and adolescent is a result of weak genes that were passed down to them by their parents. Similarly, 89.4% of the respondents report that imbecile and moron are types of neurodevelopmental disorders found in children. Further, (43.1%) of the respondents believe that supernatural power can be used to inflict neurodevelopmental disorders on children and adolescents. Of the 274 respondents in this study, 182 were Primary healthcare workers from rural/village PHCs.

**Conclusion:**

Primary healthcare workers demonstrated poor or low knowledge of neurodevelopmental disorders. On‐the‐job training (including continuing medical education), retraining, and an upgrade to the school of health curriculum are adjustments relevant to increasing the awareness and knowledge level of primary healthcare workers in identifying and managing children and adolescents with neurodevelopmental disorders.

## Summary

- The curriculum content of schools and colleges that train primary healthcare workers should include courses and extensive topics on neurodevelopmental disorders (NDDs) for students' nurses to get acquainted with issues around the identification and management of children and adolescents with neurodevelopmental disorders.
- The Ministry of Health and other stakeholders in the health profession should encourage and monitor the training and retraining of primary healthcare workers about issues concerning NDDs.
- The mental health gap action plan should be implemented across all primary healthcare centers in the country.
- The government (federal, state, and local) should empower primary healthcare centers through infrastructural development to aid assessments and management of NDDs.
- Cultural beliefs and traditions are still strongly rooted in understanding disability; therefore, collaboration and education of local authorities, religious leaders, and families need to be strengthened.

**Abbreviations**
ADHD: attention deficit hyperactivity disorder
ASD: autism spectrum disorder
CAMH: child and adolescent mental health
CHEWs: community health extension workers
CHOs: community health officers
NDDS: neurodevelopmental disorders
PHCs: primary healthcare centers

## 1 Introduction

Neurodevelopmental disorders (NDDs) apply to a group of disorders that cause some form of impairment or disruption in daily functioning due to the abnormal development of the brain [1]. One major characteristic of these NDDs is that they start in childhood, before puberty [2]. They cause difficulties in children's emotional, behavioral, social, learning, communication, motor, and physical health. The global burden of NDDs is substantial, affecting millions of children and families worldwide [3]. However, the impact is disproportionately felt in low‐ and middle‐income countries (LMICs), where limited resources, inadequate infrastructure, and lack of trained healthcare professionals exacerbate the challenges of diagnosis, treatment, and support for individuals with NDDs [4, 5]. Intellectual disability (ID), attention deficit hyperactivity disorder (ADHD), autism spectrum disorder (ASD), specific learning difficulties, neurodevelopmental motor disorders, and communication disorders are a few examples of NDDs [6, 7]. They tend to show consistent difficulties across life spans compared to the remitting and relapsing pattern that commonly characterizes mood disorders and schizophrenia [8]. Despite the elaborate literature on specific disorders in developed countries, there is little research on NDDs in LMICs.

Within the context of LMICs, Sub‐Saharan Africa (SSA) presents a particularly challenging landscape for addressing the needs of individuals with NDDs. Limited access to healthcare services, coupled with widespread poverty, food insecurity, and infectious diseases, creates a complex environment where NDDs are often overshadowed by more pressing health priorities [9]. Stigma and cultural beliefs surrounding mental health contribute further to understanding the true prevalence and impact of NDDs in the region [10]. Moreover, the scarcity of qualified child and adolescent mental health (CAMH) professionals, including psychiatrists, psychologists, and therapists, creates a critical bottleneck in the provision of specialized care and support [11].

Despite the increasing acknowledgment of NDDs as a public health priority in SSA [12], significant gaps remain in our understanding of the factors contributing to their development and impact within this specific context. While some research has focused on specific NDDs, such as epilepsy or cerebral palsy [13, 14], others exploring the validity of diagnostic tools in African settings [15], there is a critical need for more comprehensive data on the prevalence and characteristics of the broader spectrum of NDDs.

Research has shown that the knowledge and awareness of NDDs such as autism, ID, and ADHD are relatively low among primary healthcare center (PHC) workers working at various healthcare centers in Nigeria [16, 17]. Globally, about 10%–20% of children and adolescents present with emotional health and behavioral difficulties, leading to functional impairment – a large number are likely to engage PHC centers, thus it is important to improve the expertise of PHC workers through adequate trainings on NDDs [18]. Primary healthcare workers require a significant understanding and awareness of NDDs, their understanding of NDDs often supports early intervention and prevents misdiagnosis within the local community. Primary healthcare workers play vital roles in recognizing NDDs early and therefore are able to prevent diagnostic delays – these gaps if unattended to might lead to poor health outcomes for children and adolescents [19].

Furthermore, there is a meager access rate to primary healthcare services by children with disabilities when compared with children in the general population [20]. Consequently, research reveals that PHC workers in LMICs demonstrate poor understanding of some NDDs, coupled with misconceptions, salient misunderstandings and a significant lack of knowledge and awareness of NDDs [21, 22, 23]. It is reported that there exists a massive gap in the implementation of integrating mental healthcare into the primary healthcare system in Nigeria [24]. This gap in knowledge and awareness hinders the development of effective strategies to improve the early detection and intervention for NDDs in SSA, ultimately impacting the quality of life for affected children and families. Most mental health problems, including NDDs, are still being attended to at the tertiary level of the healthcare system in Nigeria [4]. Therefore, in designing programs to raise the community level of awareness about NDDs in Sub‐Saharan Africa, it is logical to use primary healthcare workers as a primary contact point for the education of the general public [16]. This can only be achieved when healthcare workers are deepened in their knowledge of the causes, symptoms or signs, clinical presentations and features of NDDs.

The study is relevant to CAMH in Africa, as it serves as a resource towards understanding NDDs. In the recommendation for an improved curriculum, continuing medical education, and training of primary healthcare workers, this study is logically positioned to facilitate such hitherto. Since there is a dearth of data concerning the prevalence of NDDs in Sub‐Saharan Africa [21, 25]; the goal of the study is to contribute to research data on the prevalence of NDDs in Sub‐Saharan Africa. The study also aims to contribute to the literature on the knowledge of primary healthcare workers as well as other general healthcare practitioners in the child and adolescent development field in Nigeria.

## 2 Methods

### 2.1 Study Location

The Federal Capital Territory, Abuja, is the capital city of Nigeria and is in the middle belt geo‐political zone of Nigeria. Abuja replaced Lagos as the capital city of Nigeria on the 12th of December 1991. According to the United Nations, Abuja grew by 139.7% between 2000 and 2010, making it the fastest‐growing city in the world. Although no recent census has been conducted in Nigeria since 2006, the World Population Review reports Abuja's population is estimated at 4,025,740 people in 2024. The original inhabitants of Abuja are the Gbagyi (Gwari), Bassa, Gwandara, Gade, Dibo, Nupe, and Koro. The common languages used for communication are English, Hausa, and the Nigerian Pidgin language. Many of the occupants in Abuja are either Christians or Muslims. This study was carried out in the Abuja Municipal Area Council (AMAC), the largest commercial region with 6 districts in Abuja. There are about 50 PHCs in AMAC supervised by the National Primary Health Care Developmental Agency (NPHCDA). The population of AMAC as a metropolitan area is about 1,960,000, a 5.67% increase from 2020. This maintains a steady increment in the population of individuals in the area.

### 2.2 Ethical Approval

Ethical approval and clearance for the study was obtained from the Research and Ethical Committee of the NPHCDA (FHREC/2021/01/96/12‐08‐21). Written informed consent was obtained from the participating primary healthcare workers after detailed explanations about the study objectives.

### 2.3 Study Design, Participants, and Sampling Method

This study employed a cross‐sectional study design to elicit responses from the respondents using questionnaires. The study population was 274 primary health care workers comprising nurses, midwives, community health officers (CHOs), and community health extension workers (CHEWs) working at the various urban and rural PHCs in AMAC, FCT, Abuja. Those with the qualifications of CHOs and CHEWS are considered non‐nursing degree respondents. Seventeen urban and rural PHCs were involved in this study. Both the PHCs and the participants were randomly selected using the ballot system.

### 2.4 Inclusion Criteria

a. Nurses, Midwives, CHOs, and CHEWs who will give their consent.
b. Nurses, Midwives, CHOs, and CHEWs who have been working in a PHC from 6 months to 35 years because these categories of healthcare workers are still in active service at this period.

### 2.5 Exclusion Criteria

a. Nurses, midwives, CHOs, and CHEWs who will not be on duty post at the time of data collection.
b. Nurses, Midwives, CHOs, and CHEWs who are 1 year closer to retirement by the federal government work standard for retirement for health workers in Nigeria. This category of healthcare workers is excluded from this study because they may be occupying managerial positions, preparing to retire from service and as such will likely have less active contact with patients (children and adolescents). Although the authors are aware that their inclusion would have probably provided depth to the findings of the research.

### 2.6 Materials

#### 2.6.1 Socio‐Demographic Questionnaire

A social demographic questionnaire by Omigbodun et al. [26] was adapted for use in this study. It asked questions about name, age, ethnicity, qualification, marital status, religion, career, gender, year(s) of experience, and so on.

#### 2.6.2 Modified Knowledge and Attitude Towards Child and Adolescent Mental Disorders (MKACAMD)

This scale has been used in previous studies in Nigeria [27]. The 38‐item scale focuses on general knowledge of, and attitude towards, CAMH disorders. However, for this study, only 29‐item knowledge‐based questions were used, while the others were on attitudes. The questions on attitudes were removed because the study did not focus on the attitudes of primary healthcare workers towards children and adolescents with NDDs. The 29‐item was modified to suit questions concerning the three NDDs covered in this study (ASD, ADHD, and ID). The instrument involves a series of question statements that respondents can answer under three options, including “agree,” “disagree,” and “I don't know.” The correct option is awarded as “1” while the wrong option is awarded as “0.” Furthermore, all the knowledge items were summed up to create a knowledge variable, which was further categorized using the GRADE approach (low, fair, and high) [28].

### 2.7 Procedure

Seventeen PHCs from 50 PHCs in the AMAC local government area of Abuja were randomly selected using the simple random technique. A ballot system was used to determine the PHC centers that were selected for the study. This was followed by a random selection of 274 workers from these 17 PHC centers. The questionnaire was distributed among the randomly selected 274 PHC workers who were on duty at the time of the visit to the 17 PHCs. The socio‐demographic questionnaire, MKACAMD, was administered to the 274 respondents in the study. The questionnaires were completed by the respondents and retrieved immediately after completion to prevent contamination or consulting study materials. The respondents receive help in cases where they do not completely understand any items on the questionnaire. All instruments used in the study were administered in English, which was the official language of communication for primary healthcare workers.

### 2.8 Data Analysis

The data obtained were entered into the computer, then cleaned and analyzed using the Statistical Package for Social Science (SPSS) Version 21. The data collected was summarized in tables, charts, and percentages. Appropriate statistical analysis (chi‐square) was used to find the association between the variables being measured.

## 3 Result

A total of 274 primary healthcare workers participated and completed the questionnaire. The majority of respondents were classified as having poor general knowledge about NDDs, with 57% (155/272) of them providing the correct response to less than 50% of the items. Respondents were aged 20–60 years (mean 39.8 ± 10.1 years). Respondents had an average working experience of 12.4 ± 6.6 years, with the majority (71.4%) having diploma (non‐nursing) qualifications obtained from colleges of health sciences. There were more males (53.6%) than females, and most of the primary healthcare facilities were from the rural/suburban areas of the Federal Capital Territory. Most of the respondents (65.8%) have not diagnosed any child with a NDD in the past. For other key demographic features of the respondents in this study, see Table 1 below.

**Table 1:** Socio‐demographic characteristics of respondents (*N* = 274).

Variable	Frequency	%
*Gender*		
Female	127	46.4
Male	147	53.6
*Age group*		
15–24	15	5.5
25–54	225	82.1
55–64	32	11.7
*Religion*		
Christian	146	53.3
Muslim	128	46.7
*Marital status*		
Single	86	31.6
Married	180	66.2
Separated/divorced	6	2.2
Distribution of respondents by PHC location*		
Urban	92	33.6
Rural	182	66.4
*Qualification*		
Non‐nurse/diploma	177	71.4
Nurse/degree	71	28.6
*Number of years of experience*		
< 10 years	82	32.4
10 years and above	171	67.6
*Previous training on NDD*		
No	102	39.2
Yes	158	60.8
*Ever diagnosed a child with NDD*		
No	171	65.8
Yes	89	34.2

*Note: N* < 274 indicates missing values; study participants were selected from 17 PHCs, 4 of which are from urban locations.

### 3.1 Knowledge of Respondents About NDDs Is Shown in the MKACAMD Questionnaire

More than half (61.7%) of the respondents agreed that NDDs are rare in children and adolescents, 15.7% do not know, and 22.6% disagreed with the statement. The majority (84.7%) of the respondents agreed that children and adolescents with NDDs are difficult to interact with, while 5.1% do not know, and 10.2% disagreed. More than half (65.7%) of the respondents agreed that children and adolescents with NDDs are likely to be violent, compared to 15.0% who do not know, and 19.3% who disagreed that children and adolescents with NDDs are likely to be violent. Slightly over half (55.8%) of the respondents agreed that NDDs are transmitted from parents to their children, compared to 17.9% who do not know, and 26.3% who disagreed with the statement. Surprisingly, a significant number (89.4%) of the respondents agreed that imbeciles and morons are types of NDDs found in children and adolescents while 5.5% do not know, and 5.1% disagreed with the question. The majority (80.3%) of the respondents agreed that simple, clear, and concise instructions should be given to manage behavior in children and adolescents with behavioral disorders, compared to 2.2% who do not know, and 17.5% who disagreed with the statement. Other knowledge variables are shown in Table 2.

**Table 2:** Knowledge of Respondents about NDDs (*N* = 274).

Items	I do not know	Disagreed	Agreed
NDDs are rare in children and adolescents	43(15.7%)	62(22.6%)	169(61.7%)
Children with NDDs are difficult to interact with	14(5.1%)	28(10.2%)	232(84.7%)
Imbecile and moron are types of NDD found in children	15(5.5%)	14(5.1%)	245(89.4%)
Children and adolescents with NDDs are likely to be violent	41(15.0%)	53(19.3%)	180(65.7%)
NDDs in children can be caused by traumatic events	4(1.5%)	81(29.6%)	189(69.0%)
Children with NDDs are from rich families, not poor families	8(2.9%)	160(58.4%)	106(38.7%)
For children and adolescents with NDDs, their families are to blame for this	17(6.2%)	170(62.0%)	87(31.8%)
The root cause of NDDs in children is a curse in the family	93(33.9%)	75(27.4%)	106(38.7%)
Children with NDDs are possessed by demons	48(17.5%)	212(77.4%)	14(5.1%)
Children and adolescents with NDDs can recover after a while	47(17.2%)	124(45.3%)	103(37.6%)
Children and adolescents with NDDs have inherited weak genes from their parents	35(12.8%)	52(19.0%)	187(68.2%)
Children and adolescents with NDDs are unpredictable	123(44.9%)	79(28.8%)	72(26.3%)
NDDs are common in our communities these days	41(15.0%)	117(42.7%)	116(42.3%)
Supernatural power can be used to inflict NDDs on a child or adolescent	104(38.0%)	52(19.0%)	118(43.1%)
NDDs in children and adolescents are caused by spiritual attacks	12(4.4%)	248(90.5%)	14(5.1%)
NDDs can be transmitted from parents to their children	49(17.9%)	72(26.3%)	153(55.8%)
Children and adolescents with NDDs have depression	68(24.8%)	23(8.4%)	183(66.8%)
Children with NDDs do not have social, academic, speech, and life skill problems, just behavioral problems	65(23.7%)	82(29.9%)	127(46.4%)
NDDs in children and adolescents can be treated just like malaria	18(6.6%)	223(81.4%)	33(12.0%)
There are a greater number of girls with NDDs than boys.	22(8.0%)	122(44.5%)	130(47.4%)
Untidy appearance in a child is a sign of NDD	64(23.4%)	78(28.5%)	132(48.2%)
Using a cane to beat or threaten a child is a way to manage their behavior when they are restless and unable to sit still	20(7.3%)	112(40.9%)	142(51.8%)
Their juvenile remand home is a good place to manage children with NDDs	49(17.9%)	137(50.0%)	88(32.1%)
PH workers can be trained to manage children with NDDs	43(15.7%)	101(36.9%)	130(47.4%)
Poor nutrition and sensitivity to some types of food are some of the causes of NDDs	30(10.9%)	59(21.5%)	185(67.5%)
Treatment of children and adolescents with mental health problems should be multidisciplinary.	29(10.6%)	116(42.3%)	129(47.1%)
Behavioral disorders in children and adolescents are best managed with medication only	43(15.7%)	146(53.3%)	85(31.0%)
Simple, clear, and concise instructions should be given to manage behavior in children and adolescents with behavioral disorders	6(2.2%)	48(17.5%)	220(80.3%)
Parents of children and adolescents with mental health problems should be involved in the treatment of their children	3(1.1%)	36(13.1%)	235(85.8%)

*Note: N* < 274 indicates missing values.

#### 3.1.1 Factors Associated With Primary Healthcare Worker's Knowledge About NDDs

Tables 3 and 4 show that out of 127 female respondents, 36.2% have low knowledge of NDDs. In contrast, 63.8% have a fair knowledge of NDDs compared to 147 male respondents, of which 42.2% show low knowledge of NDDs and 57.8% have a fair knowledge of NDDs. However, the *p*‐value indicates that there is no association between gender and knowledge of NDDs. Similarly, 73 out of 157 (46.5%) respondents who reported having had previous training on NDDs had a fair knowledge of NDDs compared to 39 out of 102 (38.2%) of those who reported not having had any previous training on NDDs. However, this association was not statistically significant (*χ* ^2^ = 1.719; *p* = 0.190). The statistics on primary health care location show that out of 181 respondents who live in rural locations, 44.8% have a low level of knowledge of NDDs, while 55.2% have a fair knowledge of NDDs compared to those who live in urban areas, where 29.0% of the respondents show a low level of knowledge of NDDs and 71% have a fair knowledge of NDDS with a statistically significant association of 0.01 with PHC location. Observation around religion shows that 45.9% of Christians demonstrate low knowledge of NDDs, while 54.1% have a fair knowledge of NDDS compared to 32% of the Muslim worshipers who have a low level of knowledge of NDDS, and 68% of them have a fair knowledge of NDDS. However, there is a statistically significant association between religion and level of knowledge, *p*‐value 0.01. Other aspects of the factors associated with primary healthcare workers' knowledge about NDDs are shown in Tables 3 and 4.

**Table 3:** Factors associated with knowledge about NDDs (*N* = 274).

Personal characteristics	General knowledge of NDDs		Total	*χ* ^2^	*p* value
	Low, *n* (%)	Fair, *n* (%)			
*Gender*					
Female	46(36.2%)	81(63.8%)	127(100%)	1.012	0.31
Male	62(42.2%)	85(57.8%)	147(100%)		
*Age*					
15–24	5(33.3%)	10(66.7%)	15(100%)	1.331	0.51
25–54	92(40.9%)	133(59.1%)	225(100%)		
55 and above	10(31.3%)	22(68.8%)	32(100%)		
*PHC location*					
Rural	81(44.8%)	100(55.2%)	181(100%)	6.357	0.01
Urban	27(29.0%)	66(71.0%)	93(100%)		
*Religion*					
Christianity	67(45.9%)	79(54.1%)	146(100%)	5.486	0.01
Islam	41(32.0%)	87(68.0%)	128(100%)		
*Marital status*					
Ever married	72(38.7%)	114(61.3%)	186(100%)	0.097	0.75
Never married	35(40.7%)	51(59.3%)	86(100%)		
*Qualification*					
Non‐nurse/diploma	66(37.3%)	111(62.7%)	177(100%)	0.864	0.35
Nurse/degree	31(43.7%)	40(56.3%)	71(100%)		

*Note: N* < 274 indicates missing values.

**Table 4:** Factors associated with knowledge about NDDs (*N* = 274) (cont.).

Personal characteristics	General knowledge of NDDs		Total	*χ* ^2^	*p* value
	Low, *n* (%)	Fair, *n* (%)			
*Years of experience*					
< 10 years	32(39.0%)	50(61.0%)	82(100%)	0.371	0.54
10 years and above	60(35.1%)	111(64.9%)	171(100%)		
*Previously trained on NDDs*					
No	63(61.8%)	39(38.2%)	102(100%)	1.719	0.190
Yes	84(53.5%)	73(46.5%)	157(100%)		
*Ever diagnosed a child of NDD*					
No	59(34.5%)	112(65.5%)	171(100%)	2.706	0.10
Yes	40(44.9%)	49(55.1%)	89(100%)		

*Note: N* < 274 indicates missing values.

## 4 Discussion

In this study, there were more respondents, 67.6%, with more than 10 years of experience, than respondents with less than 10 years of experience, 39.2%. This contrasts with the study by Bakare et al. [16], which revealed 48.0% for respondents with more than 10 years of experience and 53.0% for respondents with less than 10 years of experience working as a nurse in the healthcare setting used in that study. This inconsistency could be a result of the difference in the inclusion criteria used in this study and those of Bakare et al. [16] because inclusion criteria play a massive role in deciding who participates in a study and who does not. For instance, primary healthcare workers with up to 30 years of experience were included in this study, whereas only healthcare workers with at least 1 year of experience were included in Bakare et al. [16]. Regardless of this inclusion criteria, both studies show that more primary healthcare workers with non‐nursing/diploma qualifications demonstrate fair knowledge in the identification of children and adolescents with NDDs. Generally, this study presents that primary healthcare workers demonstrate fair knowledge of NDDs, which is inconsistent with Newton [14], an article on NDDs in LMICs, including Sub‐Saharan Africa. As opposed to Yahya et al. [29], where boys were reported to be more affected by NDDs than girls, most respondents in this study believe that girls were more affected. This is a strong departure from international studies. Although percentages might vary based on the type of disorder, globally, boys tend to have a higher prevalence of NDDs than girls [30, 31]. The statement that poor nutrition and sensitivity to certain foods are causes of NDDs was inaccurately agreed upon by most respondents in this study. While Tatlow‐Golden et al. [32] discussed these factors, scientific/medical evidence does not typically show/support that poor nutrition and food sensitivities lead to NDDs in children and adolescents [33, 34]. The severity of some symptoms of NDDs, especially in ADHD and autism, can be influenced by food sensitivities and poor nutrition in some cases, but is not an explicit cause [35, 36]. It is believed that research is ongoing to understand the nature and causes of NDDs, however, there are complex factors under consideration, such as genetic composition, environmental influences, and neurological factors [8, 37].

For the question on whether the root cause of NDDs in families is a result of a curse, 38.7% of the respondents, being the highest response in this category, agreed that the root cause of NDDS in children and adolescents in the family is a function of a curse. The rationale for this outcome could be because more than half of the respondents in the study (66.4%) are from rural PHCs, where cultural beliefs are still deeply rooted. However, this study is corroborated by a study from Ethiopia where caregivers of children with NDDs in a rural community reported that being possessed by an evil spirit can cause neurodevelopmental disorders in families [38]. These are cultural belief systems that contradict what is obtainable in empirical research. Additionally, nearly half (46.4%) of the respondents in this study believe that children and adolescents with NDDs do not have social, behavioral, academic, speech, and life skill problems, which is a strong departure from what is stated in the Diagnostic and Statistical Manual for Mental Disorders DSM‐V [2]. This finding is strongly contrasted by studies from Löytömäki et al. [39] and Evans et al. [40]. Löytömäki et al. [39] suggested that children and adolescents with diagnosed NDDs often struggle with social‐emotional and behavioral difficulties, which ultimately affect their interaction and relationships. Similarly, Evans et al. [40] in their systematic review reveal that children and adolescents diagnosed with a NDD experience a range of problems, including behavioral and academic setbacks.

Further, a vast majority reportedly agreed that an untidy appearance is a means of identifying children and adolescents with NDDs; this is unconfounded and a departure from the features of children and adolescents with NDDs captured in the Diagnostic and Statistical Manual for Mental Disorders, fifth edition [2]. Again, as opposed to what is known of NDDs to run a steady course and being disorders to be managed and not cured [41], more than half (52.7%) of the respondents in this study agreed that children and adolescents with NDDs can recover after a certain period. It is not strange for the symptoms of children and adolescents with NDDs to improve over time, however, full recovery is broadly uncommon [42, 43] Rather, some children and adolescents may demonstrate notable developmental adjustments or progress particularly when early intervention and support is provided [44]. Nevertheless, there are variations across individuals, largely relying on factors such as the type of disorder, onset, severity, and quality of intervention received.

### 4.1 Strengths, Limitations, and Future Research

This study contributes to the increasing availability of literature from Sub‐Saharan Africa, highlighting knowledge of primary healthcare workers on NDDs, and more broadly, the study adds to the growing body of knowledge/research on developmental disorders in Africa. The study, as with many quantitative or survey studies, overlooked the qualitative approach. It would be more relevant to understand the emotions and experiences of primary healthcare workers in their daily dealings with children and adolescents with NDDs. Perhaps, the instrument used presents an oversimplification of the concept of NDDs, although with limited flexibility in terms of allowing the emergence of new insight. Due to social desirability, there exist some disparities and a lack of clarity in the responses and factors (such as previously trained on, qualification, years of experience, and location) associated with the knowledge of primary healthcare workers about children and adolescents with NDDs. However, the location played a major role in producing the poor knowledge. This may point to a number of rationales such as weak monitoring and evaluation practice within the National Primary Healthcare developmental agency, poor transfer system or rotation of primary healthcare workers within Abuja, lack of training on the job, as well as irrelevant content during training, where there is. These rationales are subject to further research.

One limitation of this study is that the knowledge assessment instrument utilized a structured response format with three options: “agree,” “disagree,” and “I don't know.” While this approach effectively minimizes guessing and provides a straightforward means of scoring, it may not fully capture the depth of respondents' understanding or their reasoning behind chosen responses. Additionally, the categorization of knowledge levels into low, fair, and high using the GRADE approach, though systematic, does not account for nuances in partial knowledge or misconceptions that could be valuable in refining training interventions. Nevertheless, this methodology remains highly beneficial in assessing primary healthcare workers' knowledge of NDDs. By offering a clear, quantifiable measure of knowledge, it facilitates the identification of specific gaps in understanding, thereby informing targeted educational and capacity‐building initiatives. The structured response format ensures consistency in data collection and allows for meaningful comparisons across different healthcare settings. Moreover, the inclusion of an “I don't know” option reduces the likelihood of inaccurate responses due to guessing, thereby enhancing the reliability of findings. Ultimately, this approach serves as a valuable tool in strengthening healthcare workers' competencies, improving early identification, and enhancing the overall quality of care for children with NDDs.

## 5 Conclusion

The results of this study show misconceptions and deficits in knowledge about NDDs among the study cohorts. Primary healthcare workers, such as those who work in the various PHCs scattered around Abuja and generally in Nigeria, are the first healthcare workers that parents and families have access to within the community setting, and it is expected of them to provide well‐rounded care and service. Primary health care workers also have the responsibility to provide adequate counseling and referrals to the families of children and adolescents with NDDs. To do this effectively, they ought to be fully equipped with deep enough knowledge about NDDs, which unfortunately is not the case, as reported in this study.

## Author Contributions

**Noah Agbo:** conceptualization, writing – original draft, data curation, formal analysis. **Yetunde Adeniyi:** supervision, writing – review and editing, conceptualization. **Onoja Matthew Akpa:** formal analysis, writing – review and editing, supervision, methodology. **Olayinka Omigbodun:** project administration, resources.

## Conflicts of Interest

The authors declare no conflicts of interest.

## Transparency Statement

The lead author, Noah Agbo, affirms that this manuscript is an honest, accurate, and transparent account of the study being reported; that no important aspects of the study have been omitted; and that any discrepancies from the study as planned (and, if relevant, registered) have been explained.

## Data Availability

The data sets supporting this study's findings are available upon reasonable request to the corresponding author.
